# Surveillance for Coronaviruses in Bats, Lebanon and Egypt, 2013–2015

**DOI:** 10.3201/eid2201.151397

**Published:** 2016-01

**Authors:** Mahmoud M. Shehata, Daniel K.W. Chu, Mokhtar R. Gomaa, Mounir AbiSaid, Rabeh El Shesheny, Ahmed Kandeil, Ola Bagato, Samuel M.S. Chan, Elie K. Barbour, Houssam S. Shaib, Pamela P. McKenzie, Richard J. Webby, Mohamed A. Ali, Malik Peiris, Ghazi Kayali

**Affiliations:** National Research Centre, Giza, Egypt (M.M. Shehata, M.R. Gomaa, R. El Shesheny, A. Kandeil, O. Bagato, M.A. Ali);; The University of Hong Kong, Hong Kong, China (D.K.W. Chu, S.M.S. Chan, M. Peiris);; Lebanese University, Al Fanar, Lebanon (M. AbiSaid);; Animal Encounter, Aley, Lebanon (M. AbiSaid);; King Abdulaziz University, Jeddah, Saudi Arabia (E.K. Barbour);; American University of Beirut, Beirut, Lebanon (E.K. Barbour, H.S. Shaib);; St. Jude Children’s Research Hospital, Memphis, Tennessee, USA (P.P. McKenzie, R.J. Webby, G. Kayali)

**Keywords:** Coronavirus, bats, Middle East, viruses, zoonoses, Lebanon, Egypt, surveillance

**To the Editor:** Coronaviruses (CoVs) in bats are genetically diverse, and evidence suggests they are ancestors of Middle East respiratory virus CoV (MERS-CoV), severe acute respiratory syndrome CoV, and human CoVs 229E and NL63 (*1**–**4*). We tested several bat species in Lebanon and Egypt to understand the diversity of bat CoVs there.

Samples were collected during February 2013–April 2015. A total of 821 bats were captured live in their caves; sampled (oral swab, rectal swab, serum); and released, except for 72 bats that died or were euthanized upon capture. Lungs and livers of euthanized bats were harvested and homogenized. Caves were in proximity to human-inhabited area but not in proximity to camels.

In Egypt, we sampled 3 bat species (Technical Appendix 1). Eighty-two Egyptian tomb bats (*Taphozous perforatus*) tested negative for CoV. We also sampled 31 desert pipistrelle bats (*Pipistrellus deserti*) and detected an HKU9-like betacoronavirus (β-CoV) in the liver of 1 bat (prevalence 3.2%). From 257 specimens from Egyptian fruit bats (*Rousettus aegyptiacus*), we detected β-CoV in 18 samples from 18 different bats (prevalence 7%). A murine hepatitis virus–like CoV was detected in the lung of 1 bat. HKU9-like viruses were detected in 5 oral, 2 lung, 5 liver, and 5 rectal samples. Overall, 5.1% of the bats tested positive.

In Lebanon, we sampled 4 bat species. Four *Rhinolophus hipposideros* bats and 6 *Miniopterus schribersii* bats tested negative. One of 3 *Rhinolophus ferrumequinum* bats sampled was positive. We sampled 438 *Rousettus aegyptiacus* bats from 10 different locations and detected HKU9-like viruses in 24 rectal swab specimens (prevalence 5.5%). Overall, 5.5% of the bats tested positive.

A subset of the samples (696 samples: 516 from Egypt, 180 from Lebanon) were tested for MERS-CoV by using the specific upstream of E quantitative reverse transcription PCR; all tested negative. Serum samples from 814 bats tested negative for MERS-CoV antibodies.

Phylogenetic analysis revealed that the RNA-dependent RNA polymerase (*RdRp*) genes of viruses detected in *R. aegyptiacus* bats in Lebanon and Egypt were closely related to the *RdRp* gene of HKU9 CoV (Figure). Our viruses clustered in 3 groups: A, B, and C. Group A viruses were closely related to HKU9-10-2 virus and included viruses from Egypt. Group B included viruses from both countries and were closely related to HKU9-1 and HKU9-4 viruses. Group C also included viruses from both countries that were related to HKU9-3 and HKU9-5 viruses. The *RdRp* fragments sequenced had <90% nt similarity among groups A, B, and C. Within-group nucleotide similarity was >90%, and amino acid variability was 2%–4% (Technical Appendix 2). The phylogenetic tree of the N gene also showed proximity of the viruses detected in our study to HKU9 viruses (Technical Appendix 1). Viruses from Lebanon clustered together as did the viruses from Egypt.

**Figure:**
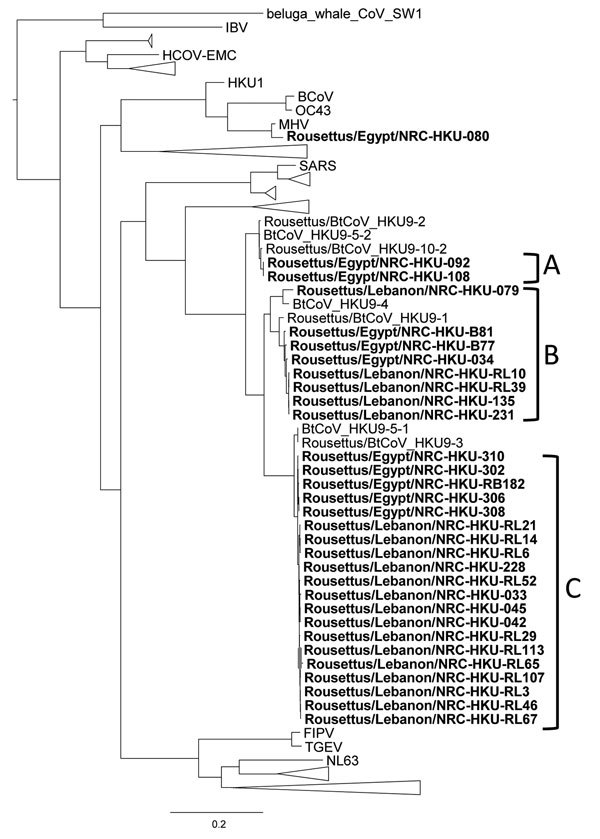
Phylogenetic tree of the coronavirus RNA-dependent RNA polymerase gene. This tree was constructed on the basis of a sequence alignment of 330 bp using the neighbor-joining method. Bold text indicates sequences found in this study. Scale bar indicates nucleotide substitutions per site.

Most of the positive samples were detected in Egyptian fruit bats. These are cave-dwelling species that inhabit regions of East Africa, Egypt, the Eastern Mediterranean, Cyprus, and Turkey (*5*). This species is a reservoir for several viruses, including Marburg, Kasokero, and Sosuga viruses (*6*–*8*). The β-CoVs HKU9 and HKU10 were detected in Chinese fruit bats (*9*). All but 1 of the detected viruses were HKU9-like. However, there was enough genetic variability within the sequenced *RdRp* fragments to suggest the circulation of at least 3 diverse groups comprising 3 different CoV species.

Our detection of CoVs in oral, rectal, lung, and liver samples suggests that CoV infection in those bats was systemic, although the bats were apparently healthy. One bat had a murine hepatitis virus–like infection. This bat was captured from a brood that inhabited the windowsills of a historic building in urban Cairo. This infection might have been a cross-species infection from mice to bats in the same habitat.

Although bats rarely come in direct contact with humans, humans can come into more frequent contact with bat urine and feces and, in the case of fruit bats, bat saliva through partially eaten fruits. Bats in the Middle East are not eaten for food but are occasionally hunted. In this study, HKU9-related viruses were detected in apparently healthy fruit bat species from Egypt and Lebanon and appear to cause systemic infection. HKU9-related viruses are not known to cause human disease. MERS-CoV was not detected in bats sampled in this study. More surveillance for bat CoVs in the Middle East is needed, and the zoonotic potential for bat-CoVs requires further study.

Technical Appendix 1 Laboratory methods.

Technical Appendix 2 Nucleotide and amino acid pairwise distances of coronaviruses found in bats, Egypt and Lebanon, 2013–2015.
