# Supplementary material for: Surveillance for Coronaviruses in Bats, Lebanon and Egypt, 2013–2015
Source: Emerg Infect Dis. 2016 Jan;22(1):148–50. doi: 10.3201/eid2201.151397 (PMC4696718; doi:10.3201/eid2201.151397)
Supplement: Technical Appendix 1 — Laboratory methods. [file 15-1397-Techapp-s1.pdf]

Article DOI: http://dx.doi.org/10.3201/eid2201.151397

Surveillance for Coronaviruses in Bats, Lebanon and Egypt, 2013–2015

Technical Appendix 1

Laboratory Methods

Screening

Viral RNA was extracted by using the Qiaamp viral RNA minikit (QIAGEN, Hilden, Germany). The RNA was eluted in 60 µL AVE buffer and was used as a template for further detection by a pan-coronavirus nested PCR targeting the *RNA-dependent RNA polymerase* (*RdRp*) gene. First-round reverse transcription PCR (RT-PCR) was conducted by using forward primer 5-GGKTG–GGAYTAYCCKAARTG-3 and reverse primer 5-TGYTGTSWRCA-RAAYTCRTG-3 and QIAGEN 1-step RT-PCR kit. A 25-µL reaction mixture contained 5 µL of 5X reaction buffer, 1 µL dNTPs, 1 µL enzyme mix, 1.5 µL (10 Pmole) forward primer, 1.5 µL (10 Pmole) reverse primer, 10 µL ddH_2O, and 5 µL of sample RNA. The PCR cycler conditions for the amplification were 50°C for 30 min (reverse transcription) then 95°C for 15 min, 45 cycle of 94°C for 15 s (denaturation), 48°C for 30 s (annealing), 72°C for 40 s (extension), then 72°C for 10 min (final extension). The PCR product was then put through a second round PCR by using a new set of primers (forward primer 5-GGTTGG-GACTATCCTAAGTGTGA-3, reverse primer 5-CCATCATCAGATAG-AATCATCAT-3) which amplify a final PCR product of 440 bp. Using Phusion High Fidelity PCR Master Mix Kit (Thermo Scientific, Waltham, MA, USA), a 25-µL reaction contained 12.5 µL of 2X phusion master mix, 1.5 µL (10 Pmole) forward primer, 1.5 µL (10 Pmole) reverse primer, 7.5 µL H_2O, and 2 µL of PCR product. The PCR cycler conditions were 98°C for 2 min then 45 amplification cycles (98°C for 15 s, 48°C for 15 s, 72°C for 30 s), then 72°°C for 2 min. The final PCR amplicons were gel purified using the

QIAquick gel purification kit (QIAGEN) and analyzed by sequencing (*1*). The *upE* quantitative reverse transcription PCR was performed as previously described (*2*).

Sequencing

The second round forward and reverse primers were to sequence the purified DNA amplicons using a BigDyeR Terminator v3.1 Cycle Sequencing Kit (Applied Biosystems, Foster City, CA, USA) according to manufacturer's instructions and were further amplified for 26 cycles at 95°C for 30 s, 50°C for 15 s, and 60°C for 4 min. The reaction product was purified by exclusion chromatography in CentriSep columns (Princeton Separations, Adelphia, NJ, USA). The recovered materials were sequenced by using a 96-capillary 3730xl DNA Analyzer (Applied Biosystems). Sequences were assembled by using SeqMan DNA Lasergene 7 software (DNASTAR, Madison, WI, USA). Sequence analysis was performed by using BioEdit 7.0 and MEGA 6.0 for multiple sequence alignment and phylogenetic tree construction of applying the neighbor-joining method with Kimura's 2-parameter distance model and 1,000 bootstrap replicates (*3*,*4*).

An RT nested PCR designed specifically for this study was used for amplifying N gene sequences of HKU9-like viruses. Reaction conditions of this assay were same as the *RdRp* assay above except for the 1-step RT-PCR with outer forward primer 5-ATGTCTGGAMGGAATAAGCCCCG-3 and inner reverse primer 5-TTATTAGGATTACGDGTGCCCAT-3, and nested PCR with inner forward primer 5-GTTCAAGCAAGAATCTGACGGTT-3 and inner reverse primer 5-ACCTTCTTCACCCACCCAGTATA-3. The expect size of the second PCR product was 400 bp.

GenBank accession nos.: KT220528–KT220562, KT368821, KT581588–KT581603.

Serology

A pseudo-particle neutralization assay was used to test bat serum against MERS-CoV as previously described (*5*).

Ethical Statement

Ethics approval was obtained from St. Jude Children's Research Hospital Institutional Animal Use and Care Committee (Memphis, TN, USA). Swabs and tissues were tested by RT-PCR, and positive samples were sequenced.

References

1. Chu DK, Leung CY, Gilbert M, Joyner PH, Ng EM, Tse TM, et al. Avian coronavirus in wild aquatic birds. J Virol. 2011;85:12815–20. PubMed http://dx.doi.org/10.1128/JVI.05838-11

2. Corman VM, Eckerle I, Bleicker T, Zaki A, Landt O, Eschbach-Bludau M, et al. Detection of a novel human coronavirus by real-time reverse-transcription polymerase chain reaction. Euro Surveill. 2012;17:20285. PubMed

3. Tamura K, Peterson D, Peterson N, Stecher G, Nei M, Kumar S. MEGA5: molecular evolutionary genetics analysis using maximum likelihood, evolutionary distance, and maximum parsimony methods. Mol Biol Evol. 2011;28:2731–9. PubMed http://dx.doi.org/10.1093/molbev/msr121

4. Hall TA. BioEdit: a user-friendly biological sequence alignment editor and analysis for Windows 95/98/NT. Nucleic Acids Symp Ser. 1999;41:95–8.

5. Perera RA, Wang P, Gomaa MR, El-Shesheny R, Kandeil A, Bagato O, et al. Seroepidemiology for MERS coronavirus using microneutralisation and pseudoparticle virus neutralisation assays reveal a high prevalence of antibody in dromedary camels in Egypt, June 2013. Euro Surveill. 2013;18:20574. PubMed http://dx.doi.org/10.2807/1560-7917.ES2013.18.36.20574

Technical Appendix 1 Table 1. Screening for coronaviruses in bats, Egypt, 2013–2015

Species	No bats	No. samples., type	Results	Location	Date
Taphozous perforatus	5	5 Serum<br>5 Oral<br>5 Lung<br>5 Liver		Abu-Rawwash, 13 km west of Cairo	Feb 2013
T. perforatus	52	52 Serum<br>52 Oral<br>52 Rectal		Abu-Rawwash, 13 km west of Cairo	Aug 2013
T. perforatus	25	24 Serum<br>25 Oral<br>25 Rectal		Abu-Rawwash, 13 km west of Cairo	Oct 2013
Pipistrellus deserti	29	29 Serum<br>29 Oral<br>29 Lung<br>29 Liver	1 (HKU9-like)	Abu-Rawwash, 13 km west of Cairo	Feb 2013
P. deserti	2	1 Serum<br>2 Oral<br>2 Rectal		Abu-Rawwash, 13 km west of Cairo	Oct 2013
Rousettus aegyptiacus	14	14 Serum<br>14 Oral<br>14 Lung	1 (murine hepatitis viru–like)	Cairo	Feb 2013
R. aegyptiacus	24	24 Serum<br>24 Oral<br>24 Lung<br>24 Liver	3 (HKU9-like)<br>2 (HKU9-like)<br>5 (HKU9-like)	Abu-Rawwash, 13 km west of Cairo	Feb 2013
R. aegyptiacus	5	4 Serum<br>5 Oral<br>5 Rectal	4 (HKU9-like)	Abu-Rawwash, 13 km west of Cairo	Oct 2013
R. aegyptiacus	102	101 Serum<br>102 Oral	2 (HKU9-like)	Abu-Rawwash, 13 km west of Cairo	Oct 2014
R. aegyptiacus	112	112 Serum	1 (HKU9-like)	Abu-Rawwash, 13 km west of Cairo	Nov 2014

Species	No bats	No. samples., type	Results	Location	Date
		112 Oral<br>112 Rectal			
Total	370		19 positive samples (5.14%)		

Technical Appendix 1 Table 2. Screening for coronaviruses in bats, Lebanon, 2013–2015

Species	No bats	Sample no. ,type	Result	Location	Date
Rhinolophus hipposideros	4	4 Serum<br>4 Rectal		Zgharta, North Lebanon	Oct 2013
Rhinolophus ferrumequinum	1	1 Serum<br>1 Rectal	1 (HKU9-like)	Aley, Mount Lebanon	Oct 2013
Rhinolophus ferrumequinum	2	2 Serum<br>2 Rectal		Amchit, North Lebanon	Oct 2013
Miniopterus schribersii	6	6 Serum<br>6 Rectal		Amchit, North Lebanon	Oct 2013
Rousettus aegyptiacus	50	50 Serum<br>50 Rectal	4 (HKU9-like)	Akkar, North Lebanon	Oct 2013
Rousettus aegyptiacus	1	1 Serum<br>1 Rectal	1 (HKU9-like)	Amchit, North Lebanon	Oct 2013
Rousettus aegyptiacus	51	51 Serum<br>51 Rectal	1 (HKU9-like)	Bisri, South Lebanon	Oct 2013
Rousettus aegyptiacus	34	34 Serum<br>34 Rectal	3 (HKU9-like)	Antelias, Mount Lebanon	Oct 2013
Rousettus aegyptiacus	21	21 Serum<br>21 Rectal	5 (HKU9-like)	Ras Keefa, North Lebanon	Apr 2014
Rousettus aegyptiacus	5	5 Serum<br>5 Rectal		Berqayel, North Lebanon	Jun 2014
Rousettus aegyptiacus	75	72 Serum<br>75 Rectal	7 (HKU9-like)	Tripoli, North Lebanon	Jun 2014
Rousettus aegyptiacus	34	34 Serum<br>34 Rectal	3 (HKU9-like)	Antelias, Mount Lebanon	Sep 2014
Rousettus aegyptiacus	101	101 Serum<br>101 Rectal		Bisri, South Lebanon	Jun 2014
Rousettus aegyptiacus	30	30 Serum<br>30 Rectal		Jbeil, Mount Lebanon	Jun 2014
Rousettus aegyptiacus	32	32 Serum<br>32 Rectal		Edde, Mount Lebanon	Oct 2014
Rousettus aegyptiacus	4	4 Serum<br>4 Rectal		Karm Saddeh, North Lebanon	Apr 2015
Total	451		25 positive samples (5.54%)		

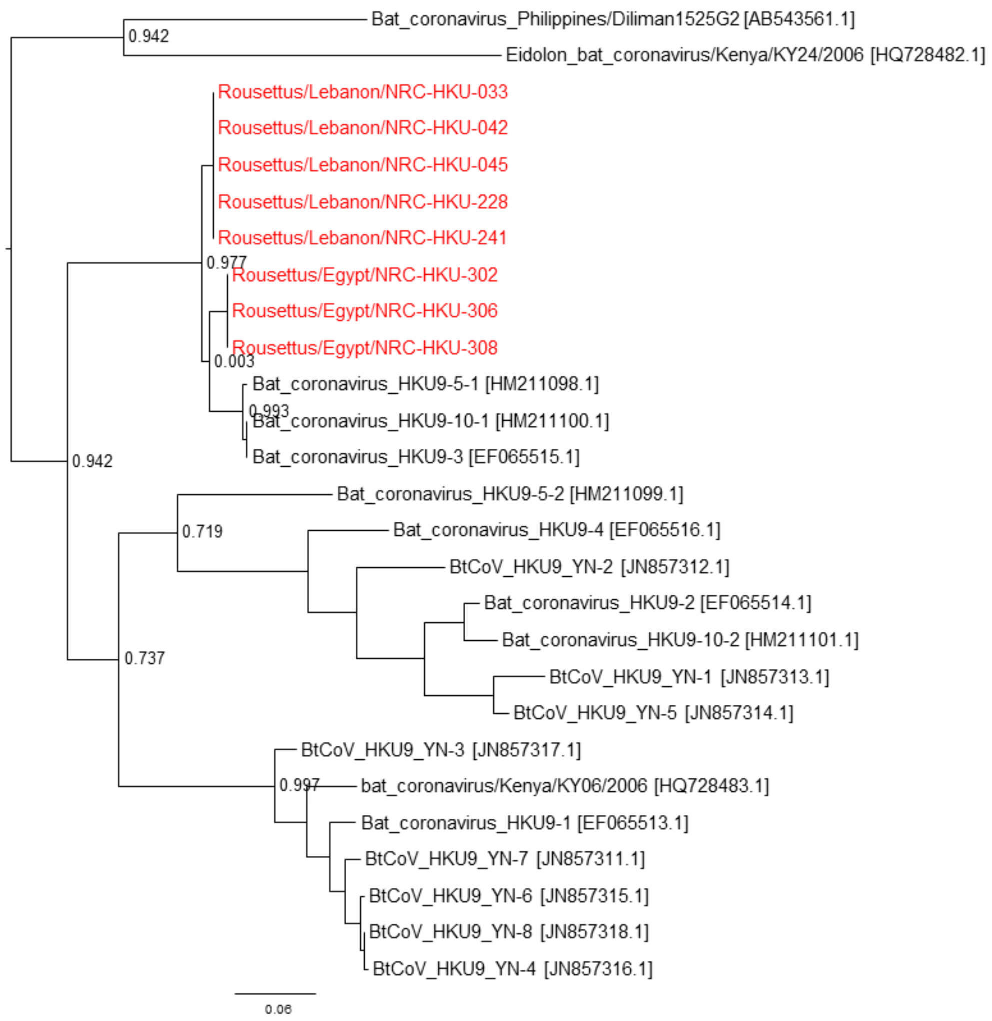

Technical Appendix 1 Figure. Phylogenetic tree of the coronavirus N gene. This tree was constructed on the basis of sequence alignment of 400 bp of the N gene and neighbor-joining method. Sequences in red are those found in this study. Scale bar indicates nucleotide substitutions per site.
